# Advancement of wound care from grafts to bioengineered smart skin substitutes

**DOI:** 10.1007/s40204-014-0030-y

**Published:** 2014-11-14

**Authors:** Robin Augustine, Nandakumar Kalarikkal, Sabu Thomas

**Affiliations:** 1 International and Interuniversity Centre for Nanoscience and Nanotechnology, Mahatma Gandhi University, Priyadarshini Hills P. O., Kottayam, 686 560 Kerala India; 2 School of Pure and Applied Physics, Mahatma Gandhi University, Priyadarshini Hills P. O., Kottayam, 686 560 Kerala India; 3 School of Chemical Sciences, Mahatma Gandhi University, Priyadarshini Hills P. O., Kottayam, 686 560 Kerala India

**Keywords:** Skin substitutes, Skin grafts, Wound healing, Angiogenesis, Smart biomaterials

## Abstract

This review gives a brief description on the skin and its essential functions, damages or injury which are common to the skin and the role of skin substitute to replace the functions of the skin soon after an injury. Skin substitutes have crucial role in the management of deep dermal and full thickness wounds. At present, there is no skin substitute in the market that can replace all the functions of skin ‘and the research is still continuing for a better alternative. This review is an attempt to recollect and report the past efforts including skin grafting and recent trends like use of bioengineered smart skin substitutes in wound care. Incorporation functional moieties like antimicrobials and wound healing agents are also described.

## Introduction

Skin is the largest organ of the body with many essential functions that would help the survival. Since it is in direct contact with the external environment which renders them highly prone to damage and/or injury. The skin plays a crucial role as a barrier against exogenous substances, pathogens and mechanical stresses. Damages to this barrier lead to loss of water and protein, and bacterial invasion to the underlying tissue. Hence, a quick regeneration or repair after an injury is necessary to avoid complications (Xiao et al. 2002). Wide range of biomaterials has been used by the medical practitioners to manage the chronic wounds (Augustine et al. 2014c). The traditional forms of wound dressings are non-resorbable gauze and/or sponge, which are made of woven or non-woven cotton mesh, cellulose or cellulose derivatives. The traditional approach sustained for over 40–45 years, which was then replaced by the advanced materials which comprise of thin films made of polyurethane that are permeable to vapour and gases. Examples of such film wound dressings include alginates, polyurethane films and hydrogels (Augustine et al. 2013a).

Many attempts have been made by the researchers to promote the regeneration of the skin. There are many successful reports on the skin regeneration upon injury by treating with allografts or autografts. For the past few decades, polymeric biomaterials were developed which can act as smart skin substitutes by performing many of the functions of skin. Presently, nanotechnology is also taken pleasure in advancing the skin substitute’s efficiency. Nanocomposites of metals such as silver and zinc that are proved to have antimicrobial activity are now incorporated with skin substitutes. Research in this field has brought in the use of biosynthetic materials and tissue-engineered living skin replacements which are now being widely termed as ‘Skin substitutes’. In the time ahead, it is expected that the researchers would find a better substitute that would render the patient a scar-free skin.

## Skin: structure and function

Skin is the largest external defence system which protects the body from pathogenic invasion. Besides the defence mechanism it is also takes part in other important functions. It serves as a mechanical barrier between the inner part of the body and the external world (Sherwood et al. 2004). The skin of an average adult body covers a surface area of approximately 2 m^2^ and weighs more than 10 % of the total body mass (Moore and Chien 1988). The skin separates the vital organs from the external environment and acts as a barrier against desiccation and various external influences. It plays a crucial role in the regulation of the body temperature and serves as a sensory organ transmitting external environmental information, such as pain and heat (Williams and Barry 1991; Barry 1983).

Microscopically, the skin is a multilayered organ composed of many histological layers. It is generally subdivided into three layers: the epidermis, the dermis and the hypodermis. The uppermost nonviable layer of the epidermis, the stratum corneum, has been demonstrated to constitute the principal barrier to percutaneous penetration (Blank 1969; Scheuplein and Blank 1971). The excellent barrier properties of the stratum corneum can be ascribed to its unique structure and composition. The viable epidermis that lies beneath is responsible for the generation of the stratum corneum. Dermis sits exactly adjacent to the epidermis and is composed of a matrix of connective tissue, which renders the skin its elasticity and resistance to deformation. The blood vessels that are present in the dermis provide the skin with nutrients and oxygen (Schaefer et al. 1996). The hypodermis or subcutaneous fat tissue supports the dermis and epidermis and provides thermal isolation and mechanical protection to the body.

## Wound and wound care

A wound is defined as a defect or break in the skin, formed due to physicochemical or thermal damage or as a result of a pathological condition. Based on the nature and repair process of wounds, they can be classified as chronic wounds or acute wounds (Boateng et al. 2008). Acute wounds are tissue injuries that heal within 8–12 weeks. The primary causes of acute wounds are mechanical injuries (friction contact between skin and hard surfaces), burns and chemical injuries. Chronic wounds heal slowly and leave serious scars. There can be different reasons that chronic wound does not heal as fast as acute wounds. The most common reasons are diabetes, infections and poor primary treatment (Boateng et al. 2008).

A wound is healthy when growth and death of microbes in the wound is balanced by the host. If the host is unable to keep the microbial growth in balance, the wound will face infection. Symptoms for an infected wound are pain, oedema, erythema, warmth and exudate. Usually wound infections are polybacterial in origin with presence of *Staphylococcus aureus* and anaerobes (*Clostridium* spp., Enterobacteriaceae, *Bacteroides fragilis* group and *Enterococcus* spp) being the most common (Fonder et al. 2008).

Wound care has evolved much in the past century from magical spells, incantations and potions to the use of sophisticated wound dressings, haemostats, hyperbaric oxygen chambers and the recent regenerative dermal substitutes. The Ebers Papyrus, circa 1500 BC, describes the use of animal grease, lint and honey as topical treatments for wounds. Despite the emergence of new therapies and the evolution of wound management, there is still a pressing need for more enhanced and efficient wound treatments. Recently, several therapies involving the administration of growth factors and stem cells to wound sites are being investigated to accelerate the wound healing process (Nie et al. 2011).

## Skin substitutes

The field of skin substitutes has been accomplished with a great deal of interest in an effort to develop the next generation of newer and better skin replacements. These dressings are made with varied combinations of synthetic and/or biologic substances. Skin substitutes are heterogeneous group of wound coverage materials that aid in wound closure and replace the functions of the skin, either temporarily or permanently, depending on the product characteristics. These substances serve as alternatives to the standard wound coverage in circumstances when standard therapies are not desirable (Shores et al. 2007). Skin substitutes are used to aid in wound closure, alleviate pain and replace the function of the skin. Skin substitutes have important roles in the treatment of deep dermal and full thickness wounds of various aetiologies (Halim et al. 2010). Treating wounds with “skin substitutes” dates back to 1880 when Joseph Gamgee described an absorbent dressing made of cotton wool sandwiched between layers of gauze (Ho 2002). A comprehensive list of skin substitutes which are currently available in the market is given in Table 1.

**Table 1:** List of currently available skin substitutes in the market

Substitute type	Commercial forms	Description	Uses
Autografts	Epicel^®^	Cultured epidermal autograft	Severe deep dermal, full thickness burns
	MySkin™	Cultured epidermal autograft	For burns, ulcers and other non-healing wounds
	Cultured skin substitutes	Cultured composite autograft	For large burns and other congenital skin disorders
	Bioseed^®^-S	Autologus keratinocyte fibrin glue suspension	Treatment of chronic leg ulcers
	CellSpray^®^	Cultured epithelilal autograft suspension	To treat superficial burns
	Stratagraft^®^	Cultured composite autograft	Burns and severe skin wounds
	Recell^®^	Autologus cell therapy device	To treat burns, scalds, traumatic wounds, scars
Allografts	Lyphoderm™	Lysate of cultured human keratinocyte	For chronic leg ulcers
	ICX-SKN	Cultured dermal allograft	To cover surgically excised partial thickness burns
	Alloderm^®^	Cadaver skin with acellular dermal matrix and intact basement membrane	For ENT/head and neck plastic reconstruction
Acellular allograft	OASIS^®^	Processed dermal xenograft	For partial and full thickness wounds and trauma wounds
Xenografts and biosynthetic grafts	Permacol™	Processed dermal xenograft	For temporary coverage of partial thickness burns
	Matriderm^®^	Bovine dermal collagen and elastin	For burns and reconstruction
	Biobrane^®^	Porcine dermal collagen bonded to semipermeable silicone membrane	To cover partial thickness burns and skin graft donor sites
	Integra^®^	Two layered skin substitute comprsisng bovine collagen and an outer silicone layer	For surgically excised deep and full thickness burns
	EZ Derm™	Porcine derived xenograft with collagen crosslinked to an aldehyde	For partial thickness wounds, donor sites, and sandwich autografts and full thickness wounds

Skin substitutes can be from humans (allografts) or animals (xenografts), or using membranes developed from natural or synthetic polymers. To date, there is no ideal skin substitute available that fulfils all the above ideal properties. Nowadays, tissue engineering and bioengineering are gearing towards the direction of creating an optimal skin substitute.

## Skin substitutes: the past and the present

The most conventional treatment that exploits the concept of skin substitution is skin grafting (Boucard et al. 2007). Skin grafting was practiced even at the time of *Koomas*, a caste recognized for pottery and tile making in India. They were now recognized as the pioneers of the skin grafting technology. The ancient Indian way was simply as pounding the skin slices obtained from a donor using a wooden slipper until it is swollen and inflamed. Research in this field has brought in the use of biosynthetic materials and tissue-engineered living skin replacements which are now being widely termed as ‘Skin substitutes’.

Three types of skin substitutes are usually referred: those consisting only epidermal equivalents, those encompassing dermal components from processed skin and those possessing distinct dermal and epidermal components, referred to as composite skins. Synthetic and natural origin polymers are now instrumental in the new strategies for the development of engineered tissue (Lanza et al. 2011). Recently, there is a huge influx of polymeric classes to be applied in biomedical field. Many are devised to stay in contact with the cells and or tissues for a long period. Biodegradable polymers are yet another class of polymers which are of great value in the biomedical application since they degrade when the functional and original tissue regenerates during healing. In the biomaterial market, the most recent advance is the availability of polymeric substitutes that are incorporated with drug and antimicrobial agents to enhance the healing or regeneration processes. Sometimes, growth factors and other extracellular matrix components are being immobilized in biopolymers, which serve as an active drug delivery system.

### Temporary skin substitutes

Temporary skin substitutes provide immediate physiological conditions for the wound closure, including protection from mechanical trauma, physical barrier to bacteria and creation of a moist wound environment (Sheridan and Moreno 2001).

### Permanent skin substitutes

Permanent skin substitutes are used to permanently achieve wound closure, replace the skin components and provide a higher quality skin replacement than the thin autologous skin graft.

### Biological skin substitutes

Biological skin substitutes act temporarily like a natural skin with the advantages of being relatively abundant in supply. These are not very expensive. The biological skin substitutes have a more intact and native extracellular matrix (ECM) structure which may allow the construction of a more natural new dermis. They also show excellent re-epithelialisation characteristics due to the presence of a basement membrane (Halim et al. 2010). The most widely used biological substitute worldwide is cadaveric skin allograft, porcine skin xenograft, amnion and cultured epithelial autografts (CEA).

#### Xenograft

**Fig. 1:**
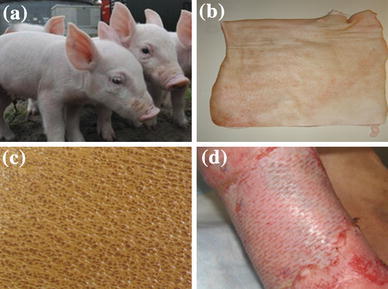
Steps in the production of porcine xenografts. Healthy pigs were selected from farm and kept under observation for several weeks (**a**), isolated skin after the sacrifice of healthy pigs (**b**), surface morphology of the skin graft harvested from pig (**c**), implanted pig xenograft in human leg burn wound (**d**)

Xenografts from various animals have been tested and tried over the centuries. Porcine skin allograft is the widely used xenograft in modern practice of burn wound care. Prior to the grafting, pig skin has been specially treated and contains only the dermis layer. A pictorial representation of the fabrication process of porcine skin xenograft is given in Fig. 1. Xenografts are mainly used for the coverage of partial thickness burns. The disadvantages include its risks of rejection and infection (Halim et al. 2010).

#### Allografts

**Fig. 2:**
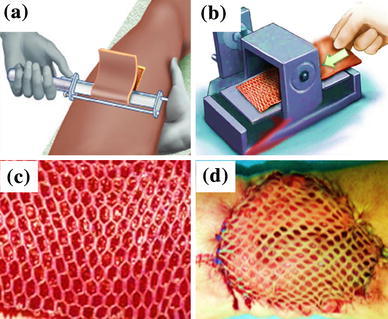
Steps in the production of allograft. Excision of skin from cadaver (**a**), meshing of isolated cadaveric skin (**b**), surface morphology of the skin graft after meshing (**c**), implanted autograft in human leg burn wound (**d**)

The cadaveric skin allograft is the most commonly used skin substitutes in burn wound management (Halim et al. 2010). Depending on the methods of processing and storage, there are two main types of cadaveric skin allografts, cryopreserved allograft and glycerol-preserved allograft (GPA). The GPA is more popular and commonly used in clinical practice (Khoo et al. 2010). A pictorial representation of the fabrication process of cadaveric skin allograft is given in Fig. 2.

#### Amnion

The amnion is a thin semi-transparent tissue found in the innermost layer of the foetal membrane. It has been used as biological dressings for burns since 1910. Because it is made from human placenta, amnion is one of the most effective substitutes to be used in healing or covering partial thickness burn wounds. Efficiency of amniotic membrane to protect wound bed as well as to reduce bacterial load in contaminated wounds is comparable with that of human skin allografts. In converse, its poor mechanical stability makes it more difficult to handle (Quinby et al. 1982).

**Fig. 3:**
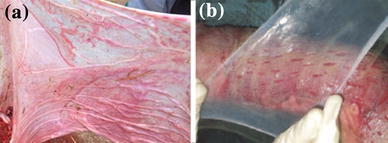
Fresh amnion collected from healthy human donor (**a**), application of cleaned amnion over split autografts (**b**)

Amnion is used to overlay the meshed autograft in addition to the application of petrolatum gauze (Fig. 3). The amnion provided good protection to the underlying meshed autograft. The non-antigenic nature, adherent quality and cost effectiveness makes amnion a promising biological dressing for meshed autografts (Lin et al. 1985).

Two varieties of amniotic membrane are mainly used as skin grafts. They are *in Toto* which is composed of amnion and chorion applied on deep burns, and *amnion alone* which composed of epithelium and basal membrane on superficial burns (Fisher 1973).

#### Cultured epithelial autografts (CEA)

Cultured epithelial autografts (CEA) are grown from patient’s own skin. Keratinocytes can be grown in culture to produce thin epithelial sheet grafts (Liu et al. 2010). The autologous keratinocytes are isolated, cultured and expanded into sheets over periods of 3–5 weeks. The use of suspension keratinocytes in fibrin glue has reduced the time for clinical use to 2 weeks (Wood et al. 2006). These grafts can act as a permanent skin replacement for patients who have deep dermal or full thickness burns. Example: Epicel^®^ marketed by Genzyme Corporation.

### Synthetic skin substitutes

Synthetic skin substitutes are fabricated from synthesized materials that are made on demand with specific characteristics required in each case. They are constructed from non-biological molecules and polymers that are not present in human skin (van der Veen et al. 2010). These constructs should be safe, stable, biodegradable and provide an adequate environment for the regeneration of tissue. Biodegradation should preferably take place after this period. An earlier product in this category is Biobrane™ (Smith & Nephew, Largo, Fla), which is still used throughout the burn community often as a temporary cover for burn and perhaps equally as often as skin donor site coverage. It is composed of two layers of silicone and a nylon mesh to which collagen is bonded. Dermagraft^®^ (Advanced BioHealing, Westport, Conn) is a synthetic product that can be used as temporary skin substitutes. Dermagraft^®^ uses either polygalactic or polyglycolic acid meshes combined with neonatal fibroblast to enhance wound healing. Other synthetic skin substitutes available in the market are Integra^®^, Apligraft^®^, Matriderm^®^, Orcel^®^, Hyalomatrix^®^ and Renoskin^®^.

Biobased materials such as chitosan, collagen, pullulan, gelatin, alginate pectin, etc. are also in the stage of in vitro or preclinical trials to use as skin substitutes (Babu et al. 2013; Mobed-Miremadi et al. 2013).

## Electrospun membranes as skin substitutes

Electrospinning is an excellent method for the fabrication of fibres with diameters from micrometre to nanometre scale (Li et al. 2013). Electrospinning technology, which can easily mass-produce thin nanofibrous membranes with good conformability, could offer a solution to the manufacture of skin substitutes. Electrospun nanofibers resemble the native topographical features of the natural extracellular matrix and may thus promote the cell’s natural functions in a biomimetic fashion. Electrospun membranes are widely used for biomedical applications like wound dressings and tissue engineering scaffolds (Augustine et al. 2014d, f). Electrospun nanofibers have various properties that make them suitable as skin substitute materials such as high oxygen permeability, variable pore size, a high surface area to volume ratio and morphological similarity to the extracellular matrix (EM) (Smith et al. 2004; Zahedi et al. 2010; Zhou et al. 2007). Various natural and synthetic polymer/polymer blends or composites have been electrospun into nanofibers to generate potential wound dressing materials. The ability to incorporate a variety of bioactive molecules (such as antimicrobials and wound healing agents) into the nanofibers can enhance the rate of wound healing.

**Fig. 4:**
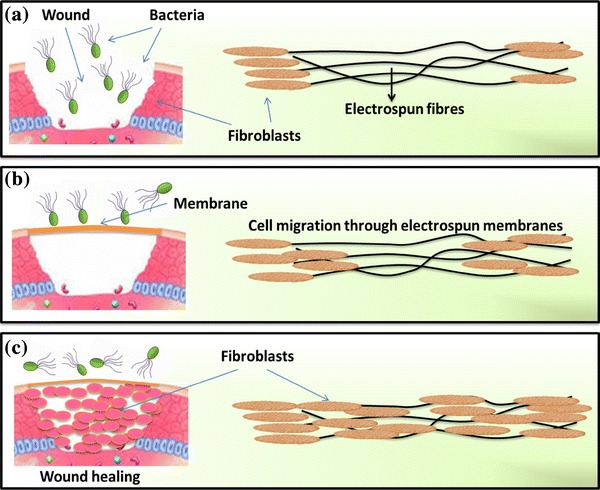
Schematic representation of the role of electrospun membranes as skin substitutes. In an open wound, bacteria will colonize and retard wound healing (**a**), while applying electrospun membranes on the wound, bacterial entry is prevented and cells guided towards the centre of the wound (**b**) and finally the wound is healed without complications and much inflammatory response (**c**)

Previous works demonstrated that electrospun membranes exhibited good capability of supporting fibroblast and/or keratinocytes attachment and proliferation with characteristic phenotypic shape and were guided to grow according to the nanofiber orientation (Fang et al. 2011). A schematic representation of the ability of electrospun membranes to act as barrier to invading bacteria and supporting fibroblast migration is shown in Fig. 4. An open wound is highly prone to bacterial colonization. Electrospun membranes will act as a physical barrier to the invading microbes and prevent the infection. We have evaluated the microbial barrier property of electrospun membranes with various pore spaces and demonstrated that these materials possessing high microbial barrier property depend on the pore spacing (Augustine et al. 2014e). Further, the fibres will support the cell migration towards the centre of the wound from the periphery. All these together contribute to the fast wound healing while using electrospun membranes as skin substitutes.

Electrospun membranes of PCL and collagen core-sheath nanofibrous membranes have shown good biocompatibility on fibroblasts (Zhang et al. 2005a, b; Zhao et al. 2007). Membranes of polymer blends were also showed good cell attachment and proliferation. Strategies like improving hydrophilicity by blending of hydrophilic polymers like polyethylene glycol (PEG) with hydrophobic polylactic acid (PLLA) nanofibers were also tried (Bhattarai et al. 2006). The presence of hydrophilic nanofibers (chitosan/PVA) in PLGA increased the absorption of culture medium during cell culture and thus promoted fibroblast attachment, proliferation, migration and infiltration in the fibre matrix (Duan et al. 2006). A previous report suggests that the proliferation of epidermal skin cells was enhanced when aligned PLLA nanofibers were used (Kurpinski et al. 2010). The relationship between fibre density and skin tissue regeneration has been investigated on electrospun gelatin skin substitutes. Cell migration was limited to the upper regions of the skin substitute if the inter-fibre distance was <5.5 μm, and the distances between 5 and 10 μm have favoured the proliferation of the cells deep into the scaffold (Powell and Boyce 2008).

Several electrospun nanofibrous membranes have been tested in epidermal tissue engineering, including those fabricated from pure natural materials or natural materials combined with synthetic polymers (Powell and Boyce 2009; Kempf et al. 2011; Franco et al. 2011). An ideal dressing should maintain a moist environment at the wound interface, allow gaseous exchange, act as a barrier to microorganisms and remove excess exudates. It should also be non-toxic, non-allergenic, non-adherent and easily removed without trauma; it should be made from a readily available biomaterial that requires minimal processing, possesses antimicrobial properties and promotes wound healing.

Hybrid materials, which combine the merits of the natural and synthetic polymeric components, have shown great advantages. One example of such hybrid scaffold is the gelatin and polycaprolactone (GT/PCL) electrospun membrane used for epidermal reconstruction (Duan et al. 2013). Dai et al. (2004) fabricated PCL/collagen composites for tissue-engineered skin substitutes and demonstrated good cell attachment and proliferation of fibroblasts and keratinocytes.

Electrospun cellulose acetate/gelatin membranes of various compositions were fabricated and their performance as a skin substitute was evaluated by Vatankhah et al. (2014). They reported that by changing the ratio of cellulose acetate and gelatin, the material can be used as tissue-engineered skin substitute with high cell adhesion properties or potential low-adherent wound dressing. Lin et al. (2013) fabricated electrospun collagen and its blends, collagen/polyvinyl alcohol (PVA) and collagen/chitosan/PVA, for skin substitute applications. They found that, compared to the skin substitute made of pure collagen, the substitutes with PVA and chitosan showed improved structural stability in aqueous solution, better tensile strength and similar or better biocompatibility in vitro.

### Advantages of electrospun membranes as skin substitutes

The wound dressing materials produced by electrospinning technology have special properties as compared to the dressings produced by conventional methods (Zhang et al. 2005a, b). Using electrospun nanofibers as skin substitutes has many advantages such as

#### Haemostasis

Electrospun membranes with small pores and high effective surface area are believed to promote haemostasis without the usage of other haemostatic agents (Wnek et al. 2003; Zhang et al. 2005a, b).

#### Exudate uptake capacity

Because of the high surface area to volume ratio of electrospun fibres, they have higher exudate uptake capacity compared to a typical film dressing therefore they absorb wound fluids more efficiently. It has been reported that the water absorption can be between 17.9 and 213 % where a standard film only absorbs 2.3 % water. Good absorptive properties of a skin substitute will help to maintain a moist environment in the wound bed (Zhang et al. 2005a, b; Williams 2007).

#### Semi-permeability

The porous structure of electrospun fibermats will provide good respiration for the cells and does not lead to wound dehydration. This will give some control of the moist environment. At the same time, the pores are so small that the fibres will protect the wound from bacterial invasion (Huang et al. 2003; Zhang et al. 2005a, b).

#### Conformability

Conformability of a wound coverage material to the wound is an important issue to be considered while designing materials intended for such applications (Queen et al. 1987). Fine fibre fabrics are easier to fit to a complicated wound with irregular architecture compared with thicker fibres, therefore electrospun fibermats will provide excellent conformability.

#### Functionability

A most desirable property of the electrospun fibermats is that they can be made bioactive by incorporating other bioactive agents. In order to minimise the infection in chronic wounds, it is important to control bioburden in a state that is not problematic to the host by delivering antimicrobial agents to the wound. A number of active agents such as drugs or other active components can be delivered to the wound from electrospun membranes in a controlled manner, which could improve the wound healing (Liang et al. 2007; Zhang et al. 2005a, b).

## Bioengineered smart skin substitutes

Smart materials are materials that have one or more properties that can be significantly changed in a controlled manner by external stimuli, such as temperature, pH, stress, moisture, electric or magnetic fields. By layman’s view, a smart biomaterial is just like a smart boy or girl who is able to manage the things very well in accordance with the circumstances. Thus, it should be able to behave in accordance with the circumstances and manage the critical situations without the effort of an external agent (Augustine et al. 2014c). Similarly, a smart skin substitutes should be able to manage the following strategies apart from the essential properties like biocompatibility, barrier properties, thermal resistance, mechanical stability, etc.

- Able to absorb excess exudate whenever it is formed on the wound bed
- Able to give optimum aeration and moisture to promote healing
- Able to deliver wound healing agents in a controlled manner
- Able to deliver antimicrobial agent at the time of infection

The first attempt in this direction is the use of synthetic degradable gels as a way to deliver cells and/or molecules in situ, the so-called smart matrix technology (Metcalfe and Ferguson 2007). The smart bioengineering concept relies on the ability of cells to sense and to adapt to their environment, and to behave in accordance with the status of the wound. Such smart skin substitutes may use different and multiple mechanisms of action for acute and chronic wounds. The success of a bioengineered skin depends on the ability of the material to stimulate the endogenous healing process (Falanga et al. 2002). Incorporation of active agents that can stimulate cell migration and proliferation is a novel approach in bioengineered skin substitutes. Vincent et al. reported that the release of cytokines and growth factors from the cells seeded in the bioengineered skin can accelerate the migration and proliferation of cells from the wound edge (Falanga et al. 2002). Bioengineered skin substitutes are manufactured by starting with a few human cells in which tissue engineers simulate the environments that allow cells to develop into viable tissue.

## Polymer nanocomposite skin substitutes with antimicrobial and wound healing properties

Application of nanoscience and nanotechnology in healthcare is rapidly evolving with the exploitation of wide range of nanomaterial with clinical relevance. Nanomaterials are materials of which a single unit is sized (in at least one dimension) between 1 and 100 nanometres (Weir et al. 2008). Nanoparticles are used in biomedical applications such as therapeutics (Kreuter and Gelperina 2008), antimicrobial agents (Raghupathi et al. 2011), transfection vectors (Tan et al. 2007) and fluorescent labels (Su et al. 2008). The nanodimension of these materials results in specific physiochemical properties that make them extremely different from their micro- or macro-scale counterparts.

Emergence of drug resistant microorganisms towards the potent antibiotics has made a lot of effort towards investigating bactericidal properties of nanoparticles (Singh et al. 2012). Most importantly, the nanoparticles tackle multiple biological pathways found in a broad spectrum of microorganisms which may require many concurrent mutations to achieve resistance against the nanoparticle’s antimicrobial activity. Extensive studies have demonstrated that the size and shape of nanoparticles have direct relationship with their antimicrobial activity (Pal et al. 2007).

Silver-containing products are most abundant in the market due to the broad spectrum antimicrobial activity of silver along with low toxicity towards mammalian cell (Jones et al. 2004; Augustine et al. 2012). Recently, biological synthesis of silver nanoparticles with more biocompatibility was successfully demonstrated by many researchers with the similar efficiency as chemical methods (Augustine et al. 2013b). Silver has been used for the treatment of microbial infections of wound since past few decades in the form metallic silver, silver nitrate and silver sulfadiazine (Dibrov et al. 2002). Zinc oxide stands next in line for their advantage silver nanoparticles such as low production cost, white appearance and UV blocking properties (Dastjerdi and Montazer 2010). The nano-zinc oxide multilayer deposited on cotton fabrics showed excellent antibacterial activity against *S*. *aureus* (Zhang et al. 2013). We have reported the fabrication of electrospun polycaprolactone (PCL) scaffolds incorporated with ZnO nanoparticles with antimicrobial and enhanced fibroblast proliferation (Augustine et al. 2014a). We also demonstrated that the ZnO nanoparticle incorporated PCL-based skin substitutes can enhance wound healing by promoting cell adhesion, migration and proliferation (Augustine et al. 2014b).

## Revascularization through skin substitutes

Rapid vascularization is essential to ensure the success of skin substitutes. Current temporary wound dressings are not designed to promote angiogenesis and granulation. Limited knowledge in the physiological processes underlying vascularization of graft preventing the successful development of skin substitutes with angiogenic properties (Lindenblatt et al. 2010). Incorporation of endothelial cells (EC) has recently demonstrated to improve vascularization of skin substitutes which has been proven by preclinical and clinical success (Black et al. 1998; Kearney 2001; Supp et al. 2002; Marston 2004; Drosou et al. 2005; Greenberg et al. 2005). Seeding of the underside of the decellularized dermal skin substitutes containing human neonatal foreskin keratinocytes with human umbilical vein endothelial cells (HUVEC) prior to the transplantation produced a successful outcome (Schechner et al. 2003). Genetic manipulation of seeded HUVEC to constitutively express the antiapoptotic protein Bcl-2 enhanced the vascularization potential in the scaffold (Schechner et al. 2000; Enis et al. 2005).

Our pioneering work demonstrated that incorporation of ZnO nanoparticles can enhance angiogenesis in electrospun polycaprolactone membranes (Augustine et al. 2014d, f). Reactive oxygen species (ROS) generated by ZnO nanoparticles were able to induce angiogenesis by the upregulation of both vascular endothelial growth factor (VEGF) and fibroblast growth factor (FGF).

## Conclusion

The history of wound care spans from prehistory to modern medicine. As the technology evolved, methodology and materials used in the care of both acute and chronic wounds have attained a new pace and direction. Much success was brought out with the introduction of skin grafts where xenografts, allografts and autografts played their role skillfully in treating wounds and traumas. Amnion is also used as a skin graft to cover full thickness burn wounds as well as to overlay meshed autografts. Cultured epithelial autografts and tissue-engineered grafts were also developed as a part of the growing interest in the field of skin regeneration upon healing. Synthetic skin grafts made from biodegradable polymers have increased the precise controllability of the structure and function of the skin substitute. They provided reduced healing time with ease of drug delivery. Use of electrospun polymeric membranes as skin substitutes is the recent trend; however, most of such attempts are at the stage of in vivo or preclinical trials. Polymer nanocomposites of metals such as silver and zinc were furnished with an aim to provide antimicrobial aid against the potential infection during the healing period. Incorporation of ZnO nanoparticles in the skin substitutes had demonstrated to improve the wound healing and angiogenic property of the skin substitutes. It is anticipated that, in future, the advancements in skin substitutes will be aimed to bring a massive change in the nature of classic wound care. The future of the skin substitutes relies on the success of the present researches which are focusing on the fabrication of cell-free smart polymeric matrices which can attract the cells towards the periphery of wound bed while acting as a physical barrier to invading microbes, delivering antimicrobials, managing wound exudates, enhancing angiogenesis and eliminating scar formation. Such bioengineered smart materials may utilize nanomaterials that can stimulate cell division, cell migration and wound healing by acting at genomic or proteomic level to regulate the expression of important biomolecules involved in these events.

## References

[CR1] Augustine R, Rajarathinam K (2012) Synthesis and characterization of silver nanoparticles and its immobilization on alginate coated sutures for the prevention of surgical wound infections and the in vitro release studies. Int J Nano Dimens

[CR8] Augustine R, Rajendran R, Cvelbar U, Mozetič M, George A (2013a) Biopolymers for health, food, and cosmetic applications. Handb Biopolym Based Mater:801-849

[CR6] Augustine R, Kalarikkal N, Thomas S (2013b) A facile and rapid method for the black pepper leaf mediated green synthesis of silver nanoparticles and the antimicrobial study. Appl Nanosci:1–10

[CR5] Augustine R, Malik HN, Singhal DK, Mukherjee A, Malakar D, Kalarikkal N, Thomas S (2014). Electrospun polycaprolactone/ZnO nanocomposite membranes as biomaterials with antibacterial and cell adhesion properties. J Polym Res.

[CR3] Augustine R, Dominic EA, Reju I, Kaimal B, Kalarikkal N, Thomas S (2014). Electrospun polycaprolactone membranes incorporated with ZnO nanoparticles as skin substitutes with enhanced fibroblast proliferation and wound healing. RSC Adv.

[CR7] Augustine R, Kalarikkal N, Thomas S (2014c) Role of wound dressings in the management of chronic and acute diabetic wounds. Diabetes Mellit Human Health Care: 273

[CR2] Augustine R, Saha A, Jayachandran VP, Thomas S, Kalarikkal N (2014). Dose dependent effects of gamma irradiation on the materials properties and cell proliferation of electrospun polycaprolactone tissue engineering scaffolds. Int J Polym Mater Polym Biomater.

[CR9] Augustine R, Thomas S, Kalarikkal N (2014e) An in vitro method for the determination of microbial barrier property (MBP) of porous polymeric membranes for skin substitute and wound dressing applications. In: Tissue engineering and regenerative medicine (In press).

[CR4] Augustine R, Dominic EA, Reju I, Kaimal B, Kalarikkal N, Thomas S (2014). Investigation on angiogenesis and its mechanism using zinc oxide nanoparticles-loaded electrospun tissue engineering scaffolds. RSC Adv.

[CR10] Babu RP, O’Connor K, Seeram R (2013). Current progress on bio-based polymers and their future trends. Prog Biomater.

[CR11] Barry BW (1983) Structure, function, diseases, and topical treatment of human skin. Dermatological formulations. Percutaneous absorption. New York, Marcel Dekker Inc. p 9

[CR12] Bhattarai SR, Bhattarai N, Viswanathamurthi P, Yi HK, Hwang PH, Kim HY (2006). Hydrophilic nanofibrous structure of polylactide; fabrication and cell affinity. J Biomed Mater Res Part A.

[CR13] Black AF, Berthod F, L’heureux N, Germain L, Auger FA (1998). In vitro reconstruction of a human capillary-like network in a tissue-engineered skin equivalent. FASEB J.

[CR14] Blank IH (1969). Transport across the stratum corneum. Toxicol Appl Pharmacol Suppl.

[CR15] Boateng JS, Matthews KH, Stevens HNE, Eccleston GM (2008). Wound healing dressings and drug delivery systems: a review. J Pharm Sci.

[CR16] Boucard N, Viton C, Agay D, Mari E, Roger T, Chancerelle Y, Domard A (2007). The use of physical hydrogels of chitosan for skin regeneration following third-degree burns. Biomaterials.

[CR17] Dai NT, Williamson MR, Khammo N, Adams EF, Coombes AGA (2004). Composite cell support membranes based on collagen and polycaprolactone for tissue engineering of skin. Biomaterials.

[CR18] Dastjerdi R, Montazer M (2010). A review on the application of inorganic nano-structured materials in the modification of textiles: focus on anti-microbial properties. Colloids Surf B.

[CR19] Dibrov P, Dzioba J, Gosink KK, Häse CC (2002). Chemiosmotic mechanism of antimicrobial activity of Ag+ in Vibrio cholerae. Antimicrob Agents Chemother.

[CR20] Drosou A, Kirsner RS, Kato T, Mittal N, Al-Niami A, Miller B, Tzakis AG (2005). Use of a bioengineered skin equivalent for the management of difficult skin defects after pediatric multivisceral transplantation. J Am Acad Dermatol.

[CR21] Duan B, Yuan X, Zhu Y, Zhang Y, Li X, Zhang Y, Yao K (2006). A nanofibrous composite membrane of PLGA–chitosan/PVA prepared by electrospinning. Eur Polymer J.

[CR22] Duan H, Feng B, Guo X, Wang J, Zhao L, Zhou G, Liu W, Cao Y, Zhang WJ (2013). Engineering of epidermis skin grafts using electrospun nanofibrous gelatin/polycaprolactone membranes. Int J Nanomed.

[CR23] Enis DR, Shepherd BR, Wang Y, Qasim A, Shanahan CM, Weissberg PL, Kashgarian M, Pober JS, Schechner JS (2005). Induction, differentiation, and remodeling of blood vessels after transplantation of Bcl-2-transduced endothelial cells. Proc Natl Acad Sci USA.

[CR24] Falanga V, Isaacs C, Paquette D, Downing G, Kouttab N, Butmarc J, Badiavas E, Hardin-Young J (2002). Wounding of bioengineered skin: cellular and molecular aspects after injury. J Investig Dermatol.

[CR25] Fang J, Wang X, Lin T (2011). Functional applications of electrospun nanofibers. Nanofibers—production, properties and functional applications.

[CR26] Fisher JC (1973). Amniotic membranes as a temporary wound dressing. Plast Reconstr Surg.

[CR27] Fonder MA, Lazarus GS, Cowan DA, Aronson-Cook B, Kohli AR, Mamelak AJ (2008). Treating the chronic wound: a practical approach to the care of nonhealing wounds and wound care dressings. J Am Acad Dermatol.

[CR28] Franco RA, Nguyen TH, Lee BT (2011). Preparation and characterization of electrospun PCL/PLGA membranes and chitosan/gelatin hydrogels for skin bioengineering applications. J Mater Sci Mater Med.

[CR29] Greenberg S, Margulis A, Garlick JA (2005) In vivo transplantation of engineered human skin. In: Epidermal Cells. Humana Press, pp 425–429 10.1385/1-59259-830-7:425 15502203

[CR30] Halim AS, Khoo TL, Yussof SJM (2010). Biologic and synthetic skin substitutes: an overview. Indian J Plast Surg.

[CR31] Ho WS (2002). Skin substitutes: an overview. Ann Coll Surg Hong Kong.

[CR32] Huang ZM, Zhang YZ, Kotaki M, Ramakrishna S (2003). A review on polymer nanofibers by electrospinning and their applications in nanocomposites. Composit Sci Technol.

[CR33] Jones SA, Bowler PG, Walker M, Parsons D (2004). Controlling wound bioburden with a novel silver-containing Hydrofiber^®^ dressing. Wound Repair Regen.

[CR34] Kearney JN (2001). Clinical evaluation of skin substitutes. Burns.

[CR35] Kempf M, Miyamura Y, Liu PY, Chen ACH, Nakamura H, Shimizu H, Tabata Y, Kimble RM, McMillan JR (2011). A denatured collagen microfiber scaffold seeded with human fibroblasts and keratinocytes for skin grafting. Biomaterials.

[CR36] Khoo TL, Halim AS, Saad AZ, Dorai AA (2010). The application of glycerol-preserved skin allograft in the treatment of burn injuries: an analysis based on indications. Burns.

[CR37] Kreuter J, Gelperina S (2008). Use of nanoparticles for cerebral cancer. Tumori.

[CR38] Kurpinski KT, Stephenson JT, Li S (2010). The effect of fiber alignment and heparin coating on cell infiltration into nanofibrous PLLA scaffolds. Biomaterials.

[CR39] Lanza, Robert, Robert Langer, Joseph Vacanti P (2011) Principles of tissue engineering. Academic press

[CR40] Li TT, Ebert K, Vogel J, Groth T (2013). Comparative studies on osteogenic potential of micro-and nanofibre scaffolds prepared by electrospinning of poly (ε-caprolactone). Prog Biomater.

[CR41] Liang D, Hsiao BS, Chu B (2007). Functional electrospun nanofibrous scaffolds for biomedical applications. Adv Drug Deliv Rev.

[CR42] Lin SD, Lai CS, Hou MF, Yang CC (1985). Amnion overlay meshed skin autograft. Burns.

[CR43] Lin HY, Kuo YJ, Chang SH, Ni TS (2013). Characterization of electrospun nanofiber matrices made of collagen blends as potential skin substitutes. Biomed Mater.

[CR44] Lindenblatt N, Platz U, Althaus M, Hegland N, Schmidt CA, Contaldo C, Vollmar B, Giovanoli P, Calcagni M (2010). Temporary angiogenic transformation of the skin graft vasculature after reperfusion. Plast Reconstr Surg.

[CR45] Liu J, Bian Z, Kuijpers-Jagtman AM, Von den Hoff JW (2010). Skin and oral mucosa equivalents: construction and performance. Orthod Craniofac Res.

[CR46] Marston WA (2004). Dermagraft^®^, a bioengineered human dermal equivalent for the treatment of chronic nonhealing diabetic foot ulcer. Expert Rev Med Devices.

[CR47] Metcalfe AD, Ferguson MWJ (2007). Tissue engineering of replacement skin: the crossroads of biomaterials, wound healing, embryonic development, stem cells and regeneration. J R Soc Interface.

[CR48] Mobed-Miremadi M, Nagendra RK, Ramachandruni SL, Rook JJ, Keralapura M, Goedert M (2013). Polystyrene microsphere and 5-fluorouracil release from custom-designed wound dressing films. Prog Biomaterials.

[CR49] Moore L, Chien YW (1988). Transdermal drug delivery: a review of pharmaceutics, pharmacokinetics, and pharmacodynamics. Crit Rev Ther Drug Carrier Syst.

[CR50] Nie C, Yang D, Jin X, Si Z, Jin X, Zhang J (2011). Locally administered adipose-derived stem cells accelerate wound healing through differentiation and vasculogenesis. Cell Transplant.

[CR51] Powell HM, Boyce ST (2008). Fiber density of electrospun gelatin scaffolds regulates morphogenesis of dermal–epidermal skin substitutes. J Biomed Mater Res Part A.

[CR52] Powell HM, Boyce ST (2009). Engineered human skin fabricated using electrospun collagen–PCL blends: morphogenesis and mechanical properties. Tissue Eng Part A.

[CR53] Queen D, Evans JH, Gaylor JDS, Courtney JM, Reid WH (1987). An in vitro assessment of wound dressing conformability. Biomaterials.

[CR54] Quinby WC, Jr HC, Hoover MS, Walters PT, Slavin SA, Bondoc CC (1982). Clinical trials of amniotic membranes in burn wound care. Plast Reconstr Surg.

[CR55] Raghupathi KR, Koodali RT, Manna AC (2011). Size-dependent bacterial growth inhibition and mechanism of antibacterial activity of zinc oxide nanoparticles. Langmuir.

[CR56] Schaefer H, Redelmeier TE (1996). Skin barrier: principles of percutaneous absorption.

[CR57] Schechner JS, Anjali KN, Lian Z, Martin KS, Christopher CWH, Rocio Sierra-Honigmann M, Lorber MI et al. (2000) In vivo formation of complex microvessels lined by human endothelial cells in an immunodeficient mouse. In: Proceedings of the National Academy of Sciences 97(16), pp 9191–9196 10.1073/pnas.150242297 PMC16844 10890921

[CR58] Schechner JS, Crane SK, Wang F, Szeglin AM, Tellides G, Lorber MI, Bothwell ALM, Pober JS (2003). Engraftment of a vascularized human skin equivalent. FASEB J.

[CR59] Scheuplein RJ, Blank IH (1971). Permeability of the skin. Physiol Rev.

[CR60] Sheridan RL, Moreno Carlos (2001). Skin substitutes in burns. Burns.

[CR61] Sherwood L (2004) Human physiology: from cells to systems. 6th Edition, Thomson Brooks, Stamford

[CR62] Shores JT, Gabriel A, Gupta S (2007). Skin substitutes and alternatives: a review. Adv Skin Wound Care.

[CR63] Singh G, Eadaoin MJ, James B, Timothy JM (2012) Evaluation of antibacterial activity of ZnO nanoparticles coated sonochemically onto textile fabrics. J Microbiol Biotechnol Food Sci 2, pp 106–120

[CR64] Smith LA, Ma PX (2004). Nano-fibrous scaffolds for tissue engineering. Colloids Surf B.

[CR65] Su J, Zhang J, Liu L, Huang Y, Mason RP (2008). Exploring feasibility of multicolored CdTe quantum dots for in vitro and in vivo fluorescent imaging. J Nanosci Nanotechnol.

[CR66] Pal S, Tak YK, Song JM (2007). Does the antibacterial activity of silver nanoparticles depend on the shape of the nanoparticle? A study of the gram-negative bacterium Escherichia coli. Appl Environ Microbiol.

[CR67] Supp DM, Wilson-Landy K, Boyce ST (2002) Human dermal microvascular endothelial cells form vascular analogs in cultured skin substitutes after grafting to athymic mice. FASEB J 16(8), pp 797–804 10.1096/fj.01-0868com PMC1820617 12039861

[CR68] Tan WB, Shan J, Yong Z (2007) Quantum-dot based nanoparticles for targeted silencing of HER2/neu gene via RNA interference. Biomaterials 28(8), pp 1565–1571 10.1016/j.biomaterials.2006.11.018 17161865

[CR69] van der Veen VC, van der Wal M, van Leeuwen MCE, Magda MWU, Esther Middelkoop (2010) Biological background of dermal substitutes. Burns 36(3), 305–321 10.1016/j.burns.2009.07.012 19897310

[CR1000] Vatankhah E, Prabhakaran MP, Jin G, Mobarakeh LG, Ramakrishna S (2014) Development of nanofibrous cellulose acetate/gelatin skin substitutes for variety wound treatment applications. J Biomater Appl 28(6):909–921 10.1177/0885328213486527 23640859

[CR70] Weir E, Antoin L, Aine W, Fiona R (2008) The use of nanoparticles in anti-microbial materials and their characterization. Analyst 133(7), 835–845 10.1039/b715532h 18575632

[CR71] Williams TR (2007) Fabrication and characterization of electrospun tecophilic scaffolds for gene delivery. PhD diss., University of Akron

[CR72] Williams AC, Barry BW (1991). Skin absorption enhancers. Crit Rev Ther Drug Carrier Syst.

[CR73] Wnek GE, Carr ME, Simpson DG, Bowlin GL (2003). Electrospinning of nanofiber fibrinogen structures. Nano Lett.

[CR74] Wood FM, Kolybaba ML, Allen P (2006). The use of cultured epithelial autograft in the treatment of major burn injuries: a critical review of the literature. Burns.

[CR75] Xiao WW, Herndon DN, Spies M, Sanford AP, Wolf SE (2002). Effects of delayed wound excision and grafting in severely burned children. Arch Surg.

[CR76] Zahedi P, Rezaeian I, Ranaei-Siadat SO, Jafari SH, Supaphol P (2010). A review on wound dressings with an emphasis on electrospun nanofibrous polymeric bandages. Polym Adv Technol.

[CR77] Zhang YZ, Venugopal J, Huang ZM, Lim CT, Ramakrishna S (2005). Characterization of the surface biocompatibility of the electrospun PCL-collagen nanofibers using fibroblasts. Biomacromolecules.

[CR78] Zhang Y, Chwee Teck L, Seeram R, ZM H (2005). Recent development of polymer nanofibers for biomedical and biotechnological applications. J Mater Sci Mater Med.

[CR79] Zhang D, Ling C, Di F, Guoyang WT, Xinxia Y, Yuyue C, Hong L (2013) In situ generation and deposition of nano-ZnO on cotton fabric by hyperbranched polymer for its functional finishing. Textile Res J

[CR80] Zhao P, Jiang H, Pan H, Zhu K, Chen W (2007). Biodegradable fibrous scaffolds composed of gelatin coated poly (ϵ-caprolactone) prepared by coaxial electrospinning. J Biomed Mater Res Part A.

[CR81] Zhou Y, Dongzhi Y, Xiangmei C, Qiang X, Fengmin L, Jun N (2007). Electrospun water-soluble carboxyethyl chitosan/poly (vinyl alcohol) nanofibrous membrane as potential wound dressing for skin regeneration. Biomacromolecules.

